# A systematic review and meta-analysis exploring the efficacy of mindfulness-based interventions on quality of life in people with multiple sclerosis

**DOI:** 10.1007/s00415-022-11451-x

**Published:** 2022-11-09

**Authors:** Robert Simpson, Stephanie Posa, Laura Langer, Tania Bruno, Sharon Simpson, Maggie Lawrence, Jo Booth, Stewart W. Mercer, Anthony Feinstein, Mark Bayley

**Affiliations:** 1 Toronto Rehabilitation Institute, University of Toronto, Toronto, Canada; 2 University of Glasgow, Glasgow, UK; 3 Glasgow Caledonian University, Glasgow, UK; 4 University of Edinburgh, Edinburgh, UK

**Keywords:** Mindfulness, Multiple sclerosis, Systematic review, Meta-analysis, Quality of life

## Abstract

**Background:**

Quality of life (QoL) is commonly impaired among people with multiple sclerosis (PwMS). The aim of this study was to evaluate via meta-analysis the efficacy of Mindfulness-based interventions (MBIs) for improving QoL in PwMS.

**Methods:**

Eligible randomized controlled trials (RCTs) were identified via searching six major electronic databases (MEDLINE, EMBASE, CINAHL, Cochrane Central Register of Controlled Trials, AMED, and PsycINFO) in April 2022. The primary outcome was QoL. Study quality was determined using the Cochrane Collaboration risk of bias tool. Meta-analysis using a random effects model was undertaken. Effect sizes are reported as Standardized Mean Difference (SMD). Prospero ID: 139835.

**Results:**

From a total of 1312 individual studies, 14 RCTs were eligible for inclusion in the meta-analysis, total participant *n* = 937. Most studies included PwMS who remained ambulatory. Cognitively impaired PwMS were largely excluded. Comorbidities were inconsistently reported. Most MBIs were delivered face-to face in group format, but five were online. Eight studies (*n* = 8) measured MS-specific QoL. In meta-analysis, overall effect size (SMD) for any QoL measure (*n* = 14) was 0.40 (0.18–0.61), *p* = 0.0003, *I*^2^ = 52%. SMD for MS-specific QoL measures (*n* = 8) was 0.39 (0.21–0.57), *p* < 0.0001, *I*^2^ = 0%. MBI effect was largest on subscale measures of mental QoL (*n* = 8), SMD 0.70 (0.33–1.06), *p* = 0.0002, *I*^2^ = 63%. Adverse events were infrequently reported.

**Conclusions:**

MBIs effectively improve QoL in PwMS. The greatest benefits are on mental health-related QoL. However, more research is needed to characterize optimal formatting, mechanisms of action, and effects in PwMS with more diverse social, educational, and clinical backgrounds.

**Supplementary Information:**

The online version contains supplementary material available at 10.1007/s00415-022-11451-x.

## Background

Multiple sclerosis (MS) is a chronic inflammatory neurodegenerative condition [1]. Comorbidity is highly prevalent [2]. Common symptoms include stress [3], anxiety [4], depression [5], fatigue [6], spasticity [7], pain [8], temperature sensitivity [9], cognitive difficulties [10], sleep impairment [11], bowel [12], bladder [13] and sexual dysfunction [14]. Over time, high levels of physical disability affect the majority [15]. People with MS (PwMS) face many challenges to their physical and mental well-being, identity, and social function [16], and commonly report impairment of quality of life (QoL). Fatigue, depression, cognitive difficulties, and physical disability exert the greatest detrimental effects [17, 18]. Other factors associated with lower QoL in PwMS include older age at disease onset, lower socioeconomic and educational statuses [19]. MS is expensive, both from the patient perspective and with regards to health and social care [20, 21]. ‘Intangible’ costs relating to patient suffering through symptoms contribute heavily to overall costs [22]. Rehabilitative approaches target functional outcomes and, ultimately, improving QoL [23, 24].

Quality of life is a multi-faceted construct, defined by the World Health Organisation as: ‘*an individual's perception of their position in life in the context of the culture and value systems in which they live and in relation to their goals, expectations, standards, and concerns. It is a broad ranging concept affected in a complex way by the person's physical health, psychological state, personal beliefs, social relationships, and their relationship to salient features of their environment*’ [25]. Measuring QoL in PwMS is complex; generic measures may not capture issues that matter most to PwMS and MS-specific measures have been developed [26]. However, as yet, no one measure captures all aspects of QoL or health-related QoL in PwMS [26].

Factors known to be associated with better QoL in PwMS include greater self-efficacy, self-esteem, resilience, and social support [17]. In addition, a recent systematic review reported psychological interventions, such as mindfulness and cognitive behavioral therapy (CBT), in addition to self-help and self-management, can improve QoL in PwMS; however, findings were in narrative format and meta-analysis was not possible due to intervention heterogeneity [17].

Mindfulness-based interventions (MBIs) are complex interventions [27], usually delivered in groups face-to-face, or, increasingly, online [28]. MBIs teach core meditation techniques aimed at enhancing attention, self-awareness, and emotion regulatory skills [29, 30]. There is high quality evidence for MBI effectiveness in non-MS populations for the treatment of stress [31], anxiety [32], recurrent depression [33] and chronic pain [34]. How MBIs work is incompletely understood, but in non-MS populations, benefits derive largely from reductions in distress, driven by increased present-moment (‘de-centring’) and body awareness [35], self-compassion [36], mindfulness [37], and reduced cognitive reactivity [38]. These benefits correlate with greater home practice [39]. Neurobiological mechanisms also include functional [40] and structural brain plasticity [41] as well as complex changes in neurohormonal [42] and immune profiles [43].

By contrast, MBI mechanisms in PwMS are poorly characterized and may be confounded by abnormal inflammatory mediator profile, monoamine dysfunction, neuronal injury, and network dysfunction [44, 45]. Nevertheless, MBIs effectively improve stress, anxiety, depression [46], and fatigue [47] in PwMS, suggesting their potential to improve QoL. However, no previous systematic review and meta-analysis has focused specifically on MBI efficacy for improving QoL in PwMS.

## Aim

The aim is to evaluate via meta-analysis the efficacy of MBIs for improving QoL in PwMS.

## Methods

### Protocol and registration

This study was registered in advance with the Centre for Reviews and Dissemination, Prospero ID: 139835.

### Study eligibility

We included all randomized controlled trials (RCTs) testing an MBI in PwMS of any phenotype, aged ≥ 18, reporting on QoL. MBIs had to contain ‘core’ components (i.e., mindful-breath awareness, body awareness, and movement) [29, 30].

### Search strategy

We searched six major electronic databases (MEDLINE, EMBASE, CINAHL, Cochrane Central Register of Controlled Trials, AMED, and PsycINFO) in April 2022 using medical subject headings and key words relating to mindfulness and multiple sclerosis, search syntax and Boolean operators. Search delimiters included: studies in humans, published in English language, between 1980—current (April 2022). We also searched reference lists, the gray literature and contacted relevant experts in the field. Our search strategies are available in Online Appendix 1.

### Study selection

Search results were imported into Endnote, for storage and screening. Two reviewers (“blinded for peer review”) independently assessed title/abstracts for eligibility. Three reviewers (“blinded for peer review”), then independently assessed eligibility against study, population, intervention, and outcome (SPIO) characteristics. A senior reviewer (“blinded for peer review”) was available for arbitration in the event of any disagreement over study eligibility.

### Data extraction

Three reviewers (“blinded for peer review”) independently extracted study data using the CONSORT and TIDieR checklists (Appendix 2).

### Quality appraisal

We used the Cochrane Collaboration tool [48] for assessing risk of bias (low, unclear, high) on individual outcomes (sequence generation, allocation concealment, participant blinding, personnel blinding, assessor blinding, incomplete outcomes, selective outcome reporting, any other source of bias). Based on summed individual outcomes, each study was then assigned an overall risk of bias category (low, unclear, high). Two reviewers engaged in discussion to reach consensus on overall risk of bias, when discrepancies arose.

### Primary outcome

Main outcome measures were all reported as continuous with mean, standard deviation (SD) values and the number of participants for each treatment group extracted. “Effect size” is reported as the unbiased standardized mean difference (SMD), a positive SMD indicating a finding in support of the intervention having a positive treatment effect. The SMD was calculated by difference in means between the MBI and the control group at follow-up divided by the pooled follow-up SD. Where effect estimates were reported from adjusted regression models, we extracted these as the SMD with their corresponding SD.

### Synthesis

We used the Preferred Reporting Items for Systematic Reviews and Meta-Analyses (PRISMA) checklist [49] when drawing together findings for our systematic review and meta-analysis. We used a random effects meta-regression model for deriving SMD, due to expected high levels of outcome heterogeneity (generic vs MS-specific QoL measures). We report effect estimates and 95% confidence intervals (as a measure of precision) and corresponding *p* values. We assessed heterogeneity using the *I*^2^ statistic, *I*^2^ representing the percentage of total variability in effect size estimates due to heterogeneity. An *I*^2^ of 0% indicates that all heterogeneity is due to sampling error, while an *I*^2^ of 100% suggests all variability may be attributable to studies being truly heterogeneous.

We computed Funnel plots and Egger’s test to determine asymmetry and likelihood of publication bias, with subsequent ‘trim and fill’ to assess significance of any bias. All statistical analyses were carried out using RevMan.

## Results

**Fig. 1:**
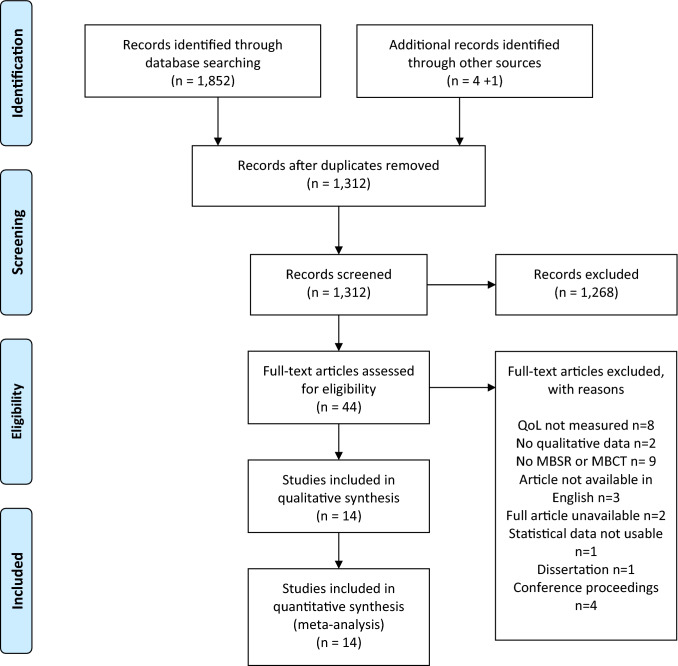
Study PRISMA flow chart

Our initial search identified 1,852 potential studies for inclusion. Following deduplication and the addition of four further studies identified via reference list searching there were 1,312 potential studies for inclusion. After title and abstract screening, 30 full text studies were reviewed, of which 14 were included in the final analyses [50–63] (Fig. 1).

### Characteristics of included studies

Eight of the 14 studies reported carrying out power calculations to determine necessary sample size [52, 55, 56, 58–60, 62, 63]; of the remaining, five did not [51, 53, 57, 58, 61] and one provided insufficient detail [54]. Studies took place across four continents, in eight different countries: three from Iran [54, 57, 63], two from Italy [55, 56], the UK [51, 53], Australia [59, 61], the USA [50, 60], and one each from Switzerland [52], Canada [62], and France [58]. Sample size ranged from 21–150. Six studies [50, 55, 56, 58, 60, 61] compared MBI against an active treatment (psychoeducation, physical activity, adaptive cognitive training, chair yoga), five usual care [51–53, 62, 63], one waitlist control [59], and in two the control condition was unclear [54, 57]. Most studies collected outcome measures thrice (pre-, post-, follow-up), but three studies were pre-post design [54, 57, 58] (Table 1).

**Table 1:** Study characteristics

Study	Country	Study design	Powered	Comparator	Sample size (n)	Study attrition (%)	Cognitive impairment exclusion criterion	QoL measure(s)	Data collection
1. Grossman et al. (2010) [52]	Switzerland	RCT	Yes	Treatment as usual	150	5%	Yes	HAQUAMS, PQOLC	Baseline, post, 6 months follow-up
2. Bogosian et al. (2015) [53]	England (UK)	RCT	No	Treatment as usual	40	5%	Yes	MSIS-29, EQ5D	Baseline, post, 3 months follow-up
3. Nejati et al. (2016) [54]	Iran	RCT	Unclear	Unclear	24	0%	No	MSQOL-54	Baseline, post
4. Simpson et al. (2017) [51]	Scotland (UK)	RCT	No	Treatment as usual	50	12%	Yes	EQ5D5L	Baseline, post, 3 months follow-up
5. Carletto et al. (2017) [56]	Italy	RCT	Yes	Psycho-education intervention	90	21%	Yes	FAMS	Baseline, post-BAM, 6 months post-BAM
6. Cavalera et al. (2019) [55]	Italy	RCT	Yes	Psycho-education intervention	139	39%	Yes	MSQOL-54	Baseline, post-, 6 months post-MBI
7. Senders et al. (2018) [50]	USA	RCT	Yes	Educational control, matched for time and attention	62	16%	Yes	SF-36 (EWS)	Baseline, mid-intervention, immediately post-, 4, 8 and 12-months post-MBI
8. Ghodspour et al. (2018) [57]	Iran	RCT	Unclear	Unclear (‘no treatment’)	30	23%	No	MSQOL-54 (MHC)	Baseline, immediately post
9. Kolahkaj et al. (2019) [63]	Iran	RCT	Yes	Treatment as usual	48	N/R	No	QoL Questionnaire	Baseline, immediately post-intervention, 2 months follow up
10. Schirda et al. (2020) [60]	USA	RCT	Yes	Active aCT group Waitlist control	61	18%	Yes	World Health Organization QoL	Baseline, immediately post-intervention, 6 month follow up
11. Torkhani et al. (2021) [58]	France	RCT	No	II + PA Control group + PA	35	II + PA: 0% MBI + PA: 47% Control + PA: 25%	No	EQ-5D-3L,	Baseline, immediately post
12. Dunne et al. (2021) [61]	Australia	RCT	No	Chair yoga Waitlist control	55	13%	Yes	MSQoL-54	Baseline, daily home practice, weekly reflective journals, post-intervention
13. Morrow et al. (2021) [62]	Canada	RCT	Yes	Standard of care	21	10%	No	SF-36	Baseline, immediately post-intervention, 3 month post
14. Sesel et al. (2022) [59]	Australia	RCT	Yes	Waitlist control	132	10%	Yes	HRQoL	Baseline, immediately post, 3 months follow up, 6 months follow up

*RCT* randomized controlled trial, *HAQUAMS* Hamburg quality of life questionnaire in multiple sclerosis (German), *PQOLC* Profile of health-related quality of life in chronic disorders (German), *MSIS-29* Multiple sclerosis impact scale-29, *MSQOL-54* Multiple sclerosis quality of life-54, *EQ5D5L* EuroQol, *FAMS* Functional Assessment of Multiple Sclerosis, *SF-36* Short form 36, *EWS* Emotional wellbeing subscale for SF-36, *MHC* Mental health composite for MSQOL-54, I*I* Implementation Intention, *PA* physical activity, *MBI* Mindfulness Based Intervention, *HRQol* Health Related Quality of Life, *aCT* Adaptive Cognitive Training

### Characteristics of study participants

Across the 14 RCTs there were 937 participants. Five studies reported on ethnicity, which was 87.8% “white” or “anglo-saxon/anglo-celtic” [50, 51, 53, 59, 60]. One study did not report the percentage of women [59], but most studies predominantly recruited women (total women = 621; 78%). Two studies did not report mean (SD) age, but rather, an age range of 20–50 [63], and a median age of 43 [58]. Of the remainder, mean (SD) age was 44.04 (9.1). Most studies did not report on socioeconomic status (SES), but in the five that did, most participants had a college degree or higher [50, 51, 53, 57, 59]. Most participants (*n* = 699; 74.5%) had a relapsing MS phenotype, while 128 (13.6%) had progressive disease. MS phenotype was not reported in the remainder. Where reported, disability, as measured by the Expanded Disability Status Scale (EDSS), was mostly < 6.0, indicating participants remained ambulant without a walking aid; however, one study focused solely on progressive MS, where mean (SD) EDSS was 6.5 (1.5) indicating the ability to walk for 20 m without stopping using walking aid(s) [53]. Four studies reported on comorbidity, mainly depression [55, 59–61]. One study reported comorbidity with a mean (SD) count of 2.4 (2.0) comorbidities [51]. In six studies, most participants were on disease-modifying drugs (DMDs) [50–52, 55, 62, 63]. One study only indicated “*both groups also received their routine drug treatments*” without specifying the number of participants on DMDs [63], and the remaining studies did not measure use. Antidepressant use ranged from 6 to 56%. Nine studies [50–53, 55, 56, 59–61] explicitly excluded those with cognitive impairment, while the remainder did not mention cognitive impairment as an eligibility criterion (Table 2).

**Table 2:** Participant characteristics

Study/Demographic	Grossman et al. (2010) [52]	Bogosian et al. (2015) [53]	Nejati et al. (2016) [54]	Simpson et al. (2017) [51]	Carletto et al. (2017) [56]	Cavalera et al. (2019) [55]	Senders et al. (2018) [50]	Ghodspour et al. (2018) [57]	Kolahkaj et al. (2019) [63]	Schirda et al. (2020) [60]	Dunne et al. (2021) [61]	Morrow et al. (2021) [62]	Torkhani et al. (2021) [58]	Sesel et al. (2022) [59]
Ethnicity	NR	90% British white	NR	100% British white	NR	NR	97% white	NR	NR	“white”: 72% “black”: 23% biracial: 3% Other: 2%	NR	NR	NR	Anglo-Celtic/Anglo-Saxon: 80% European:13% Asian: 2% Bicultural/Other: 5%
Number of participants (% female)	150 (80%)	40 (55%)	24 (46%)	50 (92%)	90 (71%)	139 (65%)	67 (78%)	30 (100%)	48 (100%)	61 (77%)	55 (83%)	21 (81%)	35 (80%)	132 (NR)
Mean age (SD)	47.3 (10.3)	52.2 (9.1)	32.3 (5.1)	45 (10.9)	44.6 (9.4)	42.7 (8.7)	52.94 (11.37)	36 (6.0)	“Ages 20–50”	45.7 (8.2)	48 (10.8)	36.8 (9.35)	“Median age” = 43.8	44.95 (10.2)
Socio-economic status	NR	NR	NR	Postcode derived; controlled in analyses	NR	NR	NR	NR	NR	NR	NR	NR	NR	NR
Employment status	NR	NR	NR	20 employed (40%)	59 employed (65%)	NR	NR	30% employed, 70% ‘homemakers’	NR	NR	NR	NR	NR	Full-Time: 32% Part-time: 36% Unemployed: 11% Registered disability: 11% Retired: 8%
Education status (SD)	Mean (SD) 14.1 (1.9) years of education	31 (77.5) had at least a college education	High school diploma at least	(56%) university level education	NR	11% elementary school; 52% high school; 38% university	60% college education or greater	62.5 Diploma and associate degree 33% BA or MBA	High school: 21 (44%) Bachelor 19 (40%)	16(1.6) years	NR	14.5 (2.5) years	NR	High school: 17% Graduate certificate/diploma: 34% Undergraduate degree:26% Postgraduate degree or more: 37%
Disease phenotype	RR 123 (82%) SP 27 (18%)	SP 23 (57.5%) PP 17 (42.5%)	NR	RR 40 (80%) SP 6 (12%) PP 4 (8%)	RR 74 (82%) SP 7 (8%) PP 2 (2%) PR 5 (6%)	RR 131 (93%) SP 8 (7%)	RR 41 (67%) SP 15 (25%) PP 4 (6%) UK 2 (3%)	RR 73% SP 13% PP 8.7% PR 4.3%	Multiple Sclerosis subtype NR	RR 59 (97%) PP 1 (2%) Unknown 1 (2%)	Multiple Sclerosis subtype NR	RMS 21 (100%)	RR 25 (71%) PP 3 (9%) SP 7 (20%)	RR 113(86%) PP 6 (5%) SP 5(4%) Don’t know 8 (6%)
EDSS score	Mean (SD) 3.0 (1.1)	Mean (SD) 6.5 (1.5)	NR	Mean (SD) 4.4 (1.8)	Mean (SD) 2.3 (1.7)	Median 3.0	Mean (SD) 4.6 (1.93)	NR	NR	4.35 (1.29)	NR	2.0 (0.0–4.0)	Mean 3.33	NR
Comorbidity	NR	NR	NR	Mean 2.4 (2.0); Range 0–9	NR	1 participant had severe depression	NR	NR	NR	NR	Trauma 1 (5.6%) Comorbid anxiety and depression 34 (56%)	NR	NR	Major depression 62 (47%)
Disease modifying drugs	91 (60.1%)	NR	NR	26 (52%)	NR	104 (85%)	34 (55%)	NR	“Both groups also received their routine drug treatments”	NR	NR	DMT 14 (66.7%)	NR	108 (82%)
Psychotropic medication	30 (20%)	NR	NR	23 (46%)	NR	9 (6%)	35 (56%)	NR	NR	9.82%	34%	NR	NR	47%

*NR* not reported, *RR* relapsing remitting, *SP* secondary progressive, *PP* primary progressive, *PR* primary relapsing, *DMT* N,N-dimethyltryptamine

### Intervention characteristics

Seven studies used Mindfulness-based stress reduction (MBSR) [50–52, 55, 59, 60, 63], two used modified MBSR (incorporating consciousness yoga [54] or somatic psychotherapy [56]). Two studies employed Mindfulness-based cognitive therapy (MBCT) [53, 57], while another adapted MBCT to an approach titled, “Mindfulness for MS” (M4MS) [61]. One study employed an MBI with physical activity [58], another used the Mindfulness Ambassador Program (MAP) [62]. All but two studies [54, 57] provided details on MBI instructor characteristics, which included certified MBSR teachers and clinical psychologists. Eleven studies delivered the MBI over 8 weeks [50, 51, 53–59, 61, 63] while others delivered over four [60], nine [52] and 10 weeks [62]. Three included a day retreat [50, 52, 56].

Four studies described detailed session content [50, 51, 53, 62]. Six provided week-by-week outlines [52, 54, 57, 60, 61, 63]. Two provided a general description [52, 58], one via study protocol [64]. Ten specified home practice [50–53, 56, 58–62]. Ten delivered group MBIs [50–57, 60, 62]. Five interventions were delivered in person [51, 52, 60, 62, 63], and five virtually, of which three [53, 55, 61] were live and two were asynchronous [58, 59]. The remainder of the studies were unclear in their intervention delivery modality (Table 3).

**Table 3:** Template for Intervention Description and Replication (TIDieR) checklist for intervention characteristics

Study/checklist item	Grossman et al. (2010) [52]	Bogosian et al. (2015) [53]	Nejati et al. (2016) [54]	Simpson et al. (2017) [51]	Carletto et al. (2017) [56]	Cavalera et al. (2019) [55]	Senders et al. (2018) [50]
1. Brief name	MBSR	MBCT	MBSR and Conscious Yoga	MBSR	Modified MBSR—Body Affective Mindfulness	MBSR	MBSR
2. Why? (rationale/theory/goal)	Cultivate interested, accepting, non-judgmental attitude to experience, including difficult sensations, emotions, thoughts, and behavior	Adaptation of MBSR. Focus on negative thinking, engaging low mood, changing relationship with thoughts, feelings, sensations, no longer avoiding/reacting to them automatically	Facilitate the compliance with and adaptation to medical conditions. Pay attention to being present in a non-judgmental manner	Cultivate interested, accepting, non-judgmental attitude to experience, including difficult sensations, emotions, thoughts, and behavior	Cultivation of mindful awareness, loving kindness, enrichment of listening, self-compassion, sensorimotor psychotherapy principles ‘window of tolerance’	Cultivate interested, accepting, non-judgmental attitude to experience, including difficult sensations, emotions, thoughts, and behavior	Cultivate interested, accepting, non-judgmental attitude to experience, including difficult sensations, emotions, thoughts, and behavior
3. What—Materials provided to participants	NR	Headset, webcam, Audio CDs for home practice	Leaflets for each session and home practice CDs	Course manual, home practice CDs, Book—*Full Catastrophe Living*	NR	Dedicated website with online multimedia for home practices	NR
4. What—Procedures pre session	Personal intake interview; goal planning	Screened for evidence of distress on GHQ	Personal intake interview	NR	NR	NR	Score of at least 10 on PSS
4. What—Procedures – in session	General description only—Observation of sensory, cognitive, and affective experience in lying, siting, and dynamic yoga postures	Session content reported in paper—Raisin exercise, Mindful awareness, body scan, sitting practice, 3-min breathing space, psychoeducation, cognitive exercises	Session outline reported in paper—Body awareness, raisin exercise, 3-min breathing, yoga, sitting meditation, psychoeducation on stress, mountain meditation	Session content reported in paper—Raisin exercise, Mindful breathing, body scan, mindful movement, psychoeducation	General description in trial protocol—Emphasis on sensorimotor resources: grounding, centering, self-soothing, psycho- education on stress, self-compassion, body scan, breath meditation, walking meditation, yoga exercises	General description only—Based on original MBSR protocol	Session content reported in paper—Mindful breathing, body scan, mindful movement, loving kindness, sitting meditation, push- pull exercise, psycho- education on stress
4. What—Procedures for home practice	40 min daily	10–20 min daily	NR	45 min daily	45 min daily	NR	45 min daily
4. What—Procedures – post course	Post course interviews for all participants	Post course interviews for some participants	NR	Post course interviews for some participants	NR	NR	NR
5. Who provided	Two experienced (> 9 years), certified teachers	Study author. Had completed MBI teacher training	NR	Two experienced (7.5 years), certified physician teachers	Trained clinical psychologists, used to working with PwMS	Expert MBSR trainer	Certified MBSR teacher with 16 years of experience
6. How—Mode of delivery	Group, face-to-face, 10–15 people per group	Group, via Skype, max 5 people per group	Group, 12 people per group	Group, face-to-face, 25 people per group	Group, number per group NR	Group, via Skype, average of 5 people per group	Group, number per group NR
7. Where—Intervention location	Unclear	Participant’s own homes	Unclear	NHS Centre for Integrative Care	Unclear	In patients own homes	NR
8. When and how much Recommended ‘dose’ = class time (h) + home practice recommendation (h)	9 weekly 2.5 h sessions 7 h practice day at week 6 Total dose: ~ 66 h	8 weekly hour sessions Total dose: ~ 24 h	8 weekly 2 h sessions Total dose: at least 16 h	8 weekly 2.5 h sessions Total dose: ~ 52 h	8 weekly 3 h sessions 7 h practice day Total dose: 63.34 h	8 weekly sessions (? duration) Total dose: unclear	8 weekly 2 h sessions 6-h practice day at week 6 Total dose: ~ 54 h
9. Tailoring	Exercises did not exceed level of function	Developed with PwMS. MBCT manual adapted for Progressive MS issues Mindful movement removed	NR	Developed with PwMS, informed MBSR optimization for future iteration	Protocol reports tailoring to needs of participants, but not reported in paper	Music meditations and acceptance of MS symptoms introduced	NR
10. In study modifications	NR	NR	NR	Mindful movement simplified	NR	NR	NR
11. How well—Treatment adherence	92% session attendance; Average 29.2 min home practice/day	18/19 (95%) completed > / = 4 sessions, home practice NR	NR	60% session attendance; Average 32.5 min home practice/day	NR	79% session attendance	85% attended > / = 6/8 sessions; median home practices 38 min day (range 14–80 min); only 55% practiced as assigned
Actual/estimated dose = actual class time (h) + actual home practice (h)	Actual/estimated dose: 27.1 + 24.4 = 51.4 h			Actual/estimated dose: 12 + 21.3 = 33.3 h			Actual/estimated dose: 18.7 + 27.2 = 45.9 h
12. How well—Fidelity assessment	NR	Senior clinical psychologist listened to session recordings for every session	NR	As per NIH guidance (2004) minus session observation/recording	NR	Treatment integrity monitored, but NR how	NR

Study/Checklist item	Ghodspour et al. (2018) [57]	Kolahkaj et al. (2019) [63]	Schirda et al. (2020) [60]	Morrow et al. (2021) [62]	Torkhani et al. (2021) [58]	Dunne et al. (2021) [61]	Sesel et al. (2022) [59]
1. Brief name	MBCT	MBSR	MBT	MBI	MBI + PA	M4MS; Chair yoga	MBI
2. Why? (rationale/theory/goal)	Focus on negative thinking, engaging low mood, changing relationship with thoughts, feelings, sensations, no longer avoiding/reacting to them automatically	to determine the effect on the quality of life	Practices targeting both focused attention and open monitoring	To assess whether an MBI would lessen the negative consequences of stress, mood symptoms and QOL, as well as objective markers of inflammation	Aimed at developing awareness of emotions and sensations	To work skillfully with pain, discomfort, and emotions	Aimed at reducing depressive symptoms, anxiety, fatigue, pain and HRQoL
3. What—Materials provided to participants	NR	NR	Homework and written study materials	Take-away assignment, designed to help reinforce the specific learnings, was assigned at the end of each session	Pre-recorded mindfulness sessions using TailorBuilder	‘Home practice materials’ (i.e., diaries, journals)	Meditation audio guides, interactive virtual modules
4. What—Procedures pre session	Interview to diagnose anxiety, depression, stress	Attend briefing session, demographic questionnaire at baseline	Pre-training assessment; daily, diary; self-report questionnaires; neuropsychological sessions	Demographic and clinical evaluation, primary, secondary, and exploratory outcomes at baseline	Neurological exam; demographic and clinical evaluation; intake screening and baseline questionnaires	If necessary, screened by clinical psychologist for suicidality; Baseline questionnaire	Pre-trial eligibility assessment; primary, secondary and process outcomes at baseline
4. What—Procedures—in session	Session outline reported in paper—Autopilot, coping with obstacles, mindful breathing, living in the moment, authorized presence, thoughts are not facts, self-care, application in negative mood states	Session content reported in paper—Raisin exercise, body-inspection, facing obstacle, yoga, mastering STOP technique, Identifying and accepting unpleasant experiences, moving from the intrapersonal to the interpersonal world, conflict management, managing outrage or conscious anger, planning for personal care, alleviating pain, writing autobiography	Session content reported in paper—Introduced to the construct of mindfulness, extended body scan meditative practice, mindful eating exercise, breath awareness, gentle standing/chair yoga, and mindful listening etc.	Session content reported in paper—Each week with a unique focus (e.g., paying attention; practicing gratitude; noticing emotional triggers; handling conflict; nurturing compassion), in-session guided mindfulness skills (e.g., mindful breathing, mindful listening, body scan practices). A take-away assignment, designed to help reinforce the specific learnings, was assigned at the end of each session	General description only—All practice was home practice (see below)	Session content reported in paper—M4MS: Taught participants to work skillfully with pain, discomfort, and emotions Chair Yoga: simple movements incorporating breathing and relaxation techniques Daily home practice diaries and weekly reflective journals to be completed	Session content reported in paper- All practice was home practice
4. What—Procedures for home practice	NR	NR	Engaging in the respective practices for 40 min each day for the remaining 6 days of each week	A take-away assignment, designed to help reinforce the specific learnings, was assigned at the end of each session	Listen to prerecorded sessions and follow instructions, receive weekly phone call	10 min of home practice encouraged every day for both intervention programs	Five interactive modules, Five meditation audio-guides, tele-coaching
4. What—Procedures—post course	NR	The quality-of-life questionnaire post-test and 2 months follow up	Post-training assessment session	All baseline measures repeated at post-intervention (or equivalent) and 6 months later	Questionnaire 8 weeks after randomization	post-intervention questionnaire	Post-intervention questionnaires at week 9, 3 months and 6 months post-intervention
5. Who provided	NR	Trained psychologist	Doctoral students in clinical psychology	RN with clinical and research experience with PwMS who was trained to be a MAP facilitator	No assistance for mindfulness; physical and sports activity trainer for PA	M4MS: Clinical psychologist who is certified mindfulness practitioner Chair Yoga: Registered yoga teacher	Internet adaptation created by psychologists; brief ‘tele-coaching’ calls with psychologists
6. How—Mode of delivery	Group, method of delivery unclear	In person	Group- In person (group sizes ranged from 2 to 5)	In person- group	Virtual	Virtual- via live web sessions, but sessions also recorded	Virtual
7. Where—Intervention location	NR	All the MBRS sessions were held in Ahvaz MS Society	Department of Psychology at The Ohio State University	NR	Home, place of participants choosing	Home, place of participant choosing	Home, place of participants choosing
8. When and how much	8 weekly 2 h sessions	2 h; weekly 8 weeks	4 weekly sessions; 2 h + 40 min a day for the remaining 6 days of the week	1 h; weekly; 10 weeks; take away assignment NR	10 min; 6 days a week; 8 weeks	1 h; weekly; 8 weeks; + 10 min of home practice per day	5 modules- 15 min each; 8 weeks + 5–8 brief telephone calls, 10 min each + 5 meditation guides; 30 min each; daily
Recommended ‘dose’ = class time (h) + home practice recommendation (h)	Total dose: at least 16 h	Total ‘dose’ = 16 h	Total ‘dose’ ~ at least = 8 h + 16 h = 24 h	Total ‘dose’ = 10 h	Total ‘dose’ ~ 8 h ( 1 h per week)	Total ‘dose’ ~ 8 h + 9.33 h home practice = 17.33 h total	Total ‘dose’ ~ 210 min per week × 8 weeks = 28 h total
9. Tailoring	Original MBCT protocol translated into Persian	NR	Adapted to be 4 weeks rather than 8	The research team, in partnership with MWB, adapted the Mindfulness ambassador Program for use in the PwMS (i.e., 10 weeks instead of 12 weeks)	“Adapted if required”	M4MS adapted from Mindfulness-based cognitive therapy; sessions 1 h rather than 2 Chair yoga adapted from traditional Hatha yoga	Yes- internet version adapted based on interviews with PwMS and experts in the field using co-design methodology;
10. In study modifications	NR	NR	NR	NR	NR	NR	Hatha yoga component of MBSR was omitted
11. How well-Treatment adherence	NR	2 MBSR participants lost to follow up	75% of the MBT participants attended all four weekly sessions and did homework on an average of 20.8 days	Three subjects randomized to the MBI group in the spring session missed > 2 sessions and removed from study. One subject in the MBI spring session, withdrew consent	Actual/estimates dose = 4.24 h	M4MS: Actual/estimated dose = 57% of 8 h (4.56 h) + 20 (7) home practice minutes	Actual/estimated dose = 136 min
Actual/estimated dose = actual class time (h) + actual home practice (h)		Actual/estimated ‘dose’ NR	Actual/estimated dose = 8 + 13.86 = 21.86 h	Actual/estimated ‘dose’ NR	Therefore, 53% adherence for MBI	Chair Yoga actual/estimated dose = 13% of 8 h (1.04 h) + 24(4) home practice minutes	54 participants (87%) completed at least 4/5 modules
12. How well—Fidelity assessment	NR	NR	Attendance, completion of homework and practice time monitored	NR	Weekly telephone call -detailed report concerning the session(s) was reviewed	NR	A meditation adherence questionnaire

*NR* not reported, *MBI* mindfulness-based intervention, *PA* physical activity, *M4MS* Mindfulness for Multiple Sclerosis, *MBSR* Mindfulness-Based Stress Reduction

### Treatment adherence, intervention fidelity, and study attrition

Among those studies reporting on MBI session attendance (seven studies [50–53, 55, 60, 61]), this ranged from 60 to 95%. Others reported on virtual session completion [59, 61], one reporting 90% of participants completed at least 4/5 modules [59], another stating 57% of participants attended live virtual sessions over the 8-week MBI [61]. Those reporting on home practice completion (six studies [50–52, 59–61]) reported a range of 29.2–38 min/day [50–52, 61], 136 min per week [59], or 817 min over the intervention period [60]. Six studies considered intervention fidelity [51, 53, 55, 58–60]. Study attrition ranged from 0 to 39%. One study did not report on intervention adherence, fidelity, or study attrition [63]. In one study, 33% (4/12) participants assigned to the MBI withdrew and were not included in the 6-month follow-up analysis [62].

### Outcome characteristics

The majority of included studies (*n* = 8) used MS-specific QoL measures. Four studies used the Multiple Sclerosis Quality of Life-54 (MSQOL-54) [54, 55, 57, 61], one the Hamburg Quality of Life Questionnaire in Multiple Sclerosis (HAQUAMS) [52], two the Multiple Sclerosis Impact Scale-29 (MSIS-29) [53, 59], one the Functional Assessment of Multiple Sclerosis (FAMS) [56]. Those employing generic measures used health-related QoL measures such as the EuroQol (EQ-5D) [51, 58], Short Form-36 (SF-36) [50, 62], and Profile of health-related Quality Of Life in Chronic disorders (PQOLC) [52], as well as general QoL measures such as the World Health Organization Quality of Life (WHOQoL) [60], Satisfaction With Life Scale (SWS) [60], and the Quality of Life Scale (QoLS) [63].

### Meta-analysis

#### Effect of MBIs on QoL

**Fig. 2:**
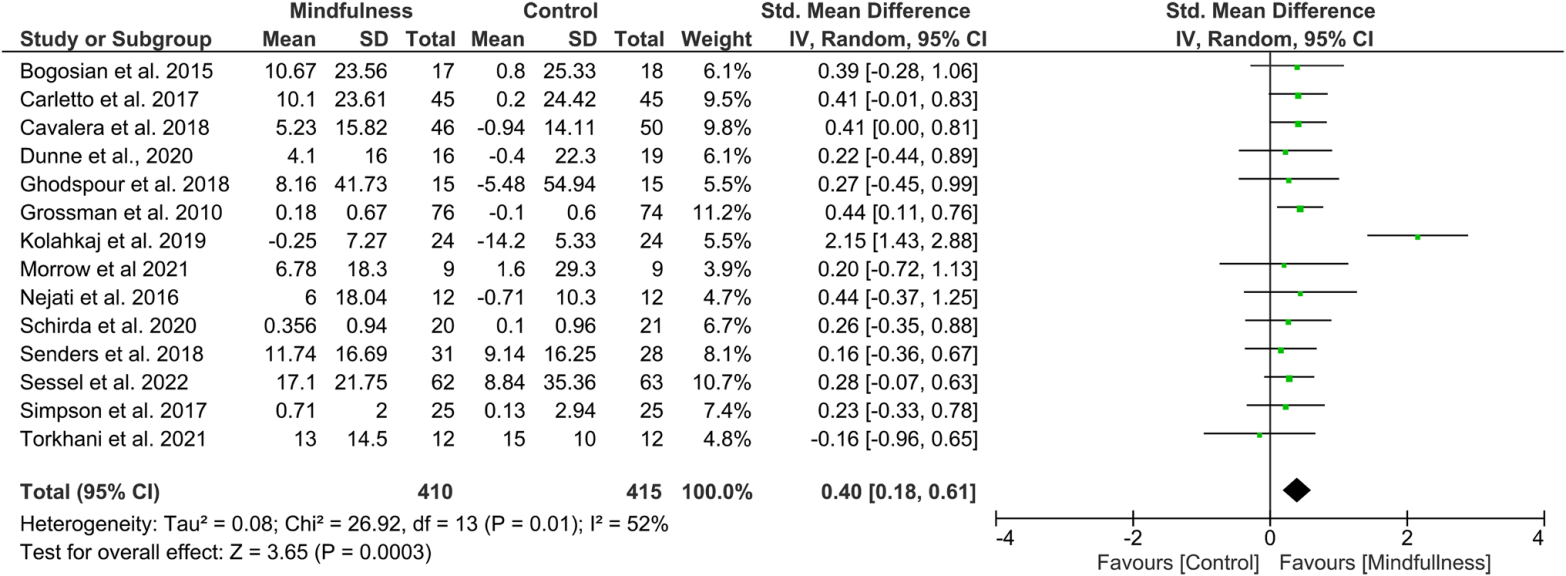
Overall meta-analysis (any QoL measure)

**Fig. 3:**
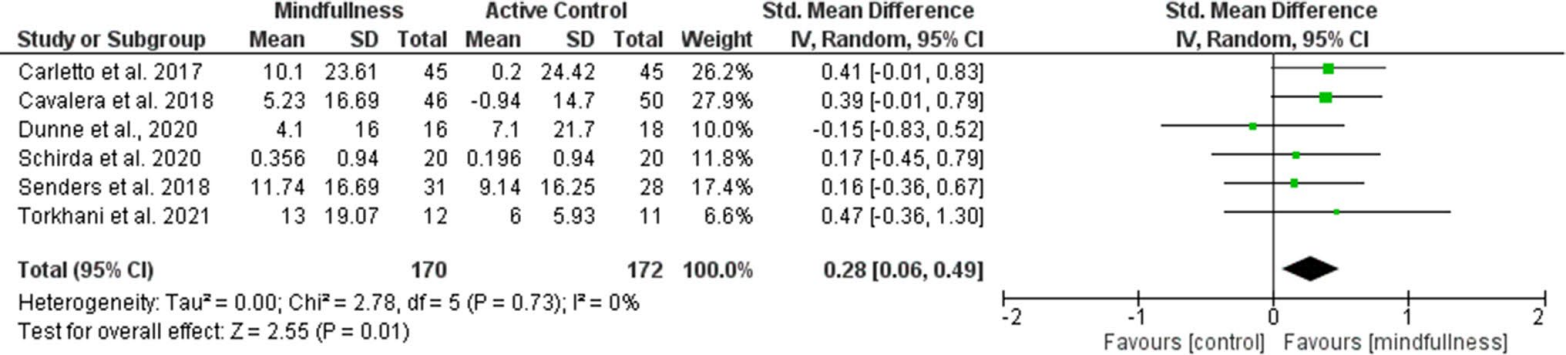
Active comparator studies only

**Fig. 4:**
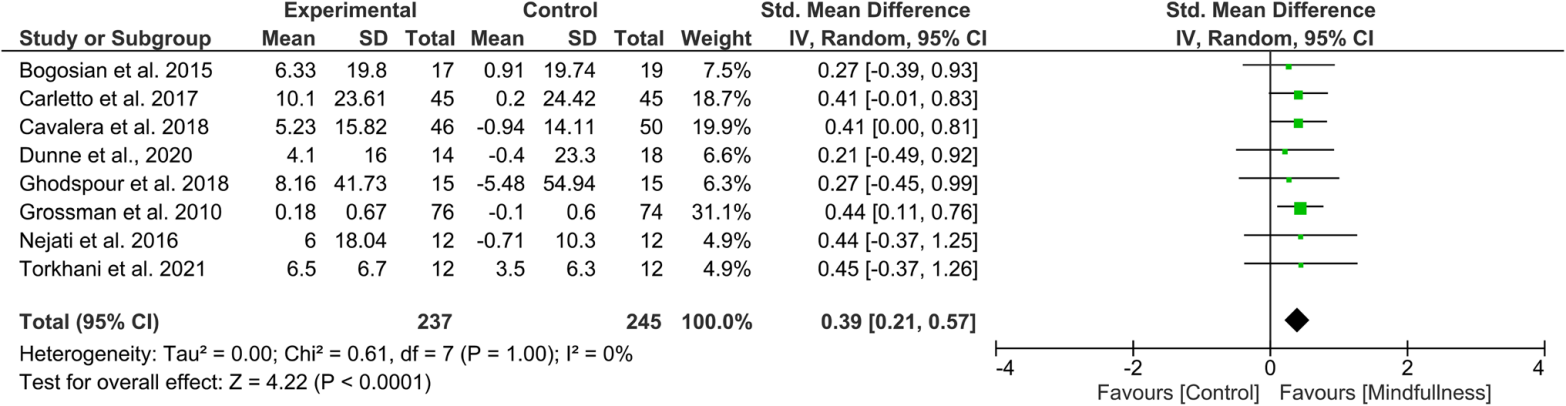
MS-specific QoL measures only

**Fig. 5:**
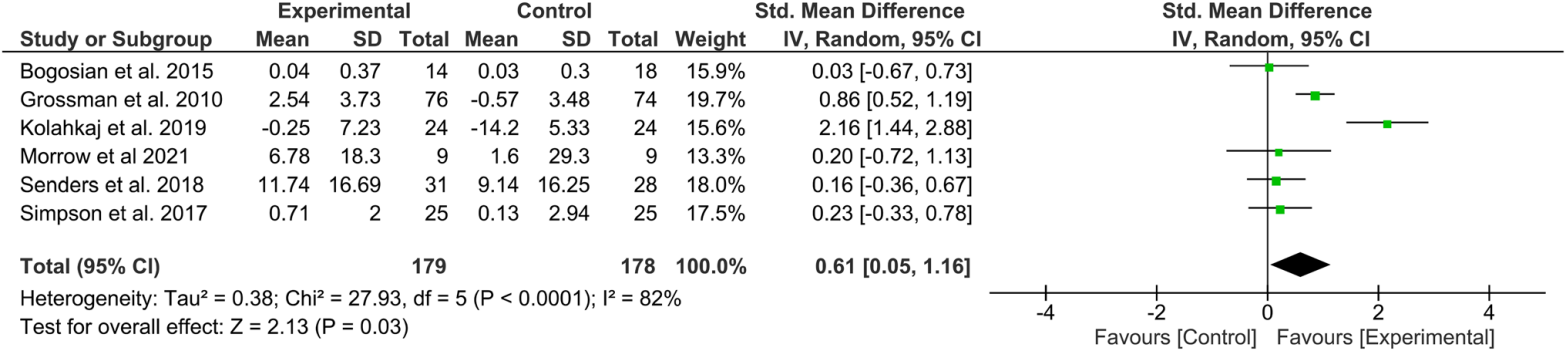
Generic QoL measures only

**Fig. 6:**
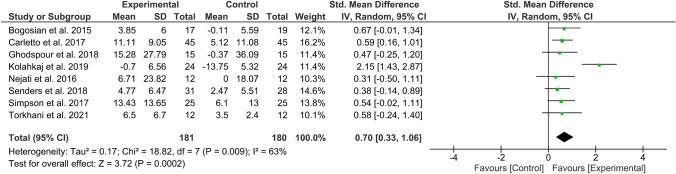
Mental QoL measures only

**Fig. 7:**
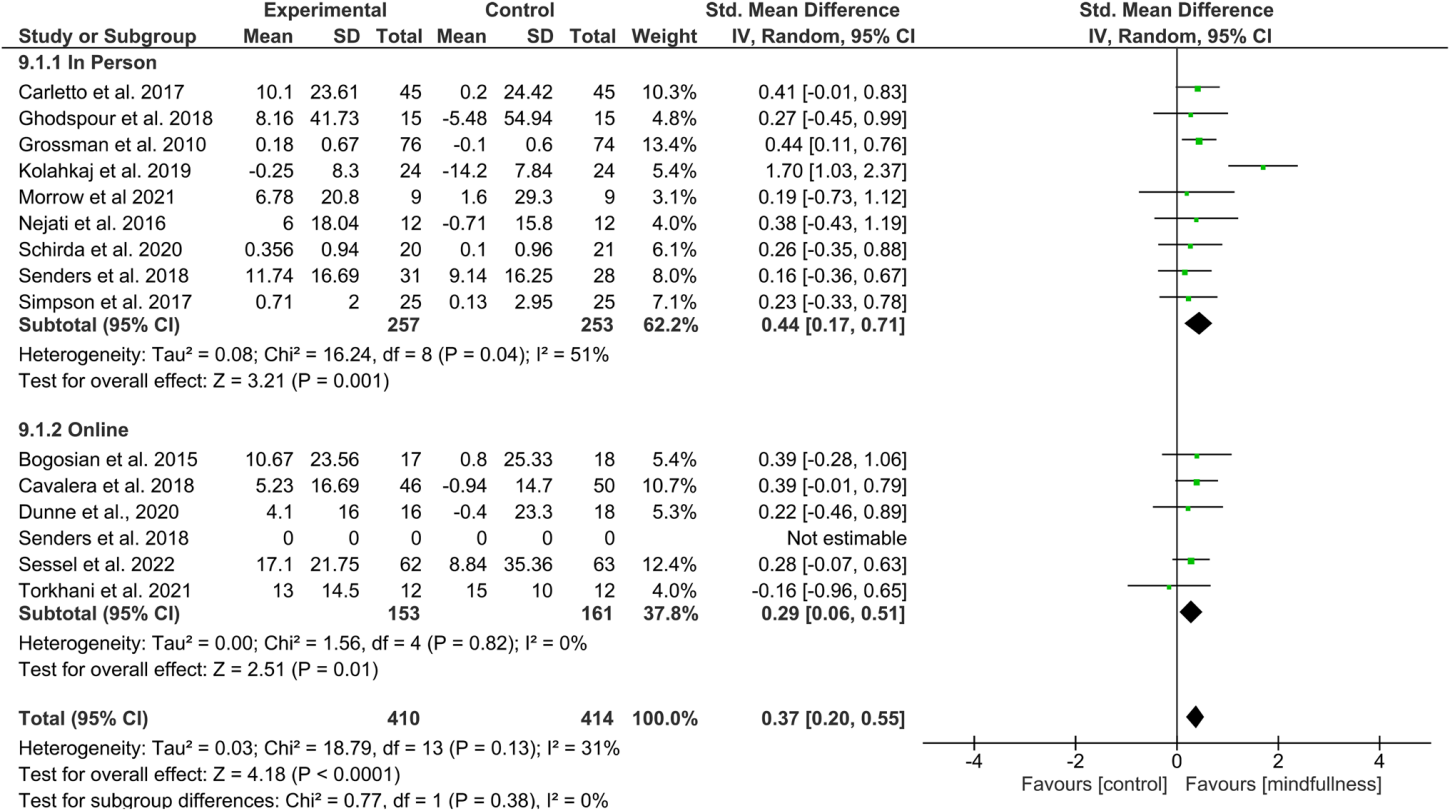
Face-to-face vs online MBI

Overall effect size (SMD) in the meta-analysis for any QoL measure (*n* = 14) was 0.40 (0.18–0.61), *p* = 0.0003; heterogeneity was moderate (*I*^2^ = 52%) (Fig. 2). When examining only those studies which included an active comparator (*n* = 6), the SMD was 0.28 (95% CI 0.06–0.49), p = 0.01, *I*^2^ = 0% (Fig. 3). SMD for MS-specific QoL measures (*n* = 8) was 0.39 (0.21–0.57), *p* < 0.0001, *I*^2^ = 0%. (Fig. 4). Among those studies using generic QoL measures (*n* = 6), SMD was 0.61 (95% CI: 0.05–1.16), *p* = 0.03, *I*^2^ = 25% (Fig. 5). MBI effect was largest on subscale measures of mental QoL (n = 8), where SMD was 0.70 (0.33–1.06), *p* = 0.0002, though heterogeneity was substantial (*I*^2^ = 63%). (Fig. 6). Face-to-face MBIs (*n* = 9) had a larger SMD 0.44 (0.17–0.71), *p* = 0.001, but with moderate heterogeneity (*I*^2^ = 51%), when compared with online MBIs (*n* = 5), SMD 0.29 (0.06–0.53), *p* = 0.01, *I*^2^ = 0%, but these differences were not statistically significant (*p* = 0.38) (Fig. 7).

### Heterogeneity and publication bias

**Fig. 8:**
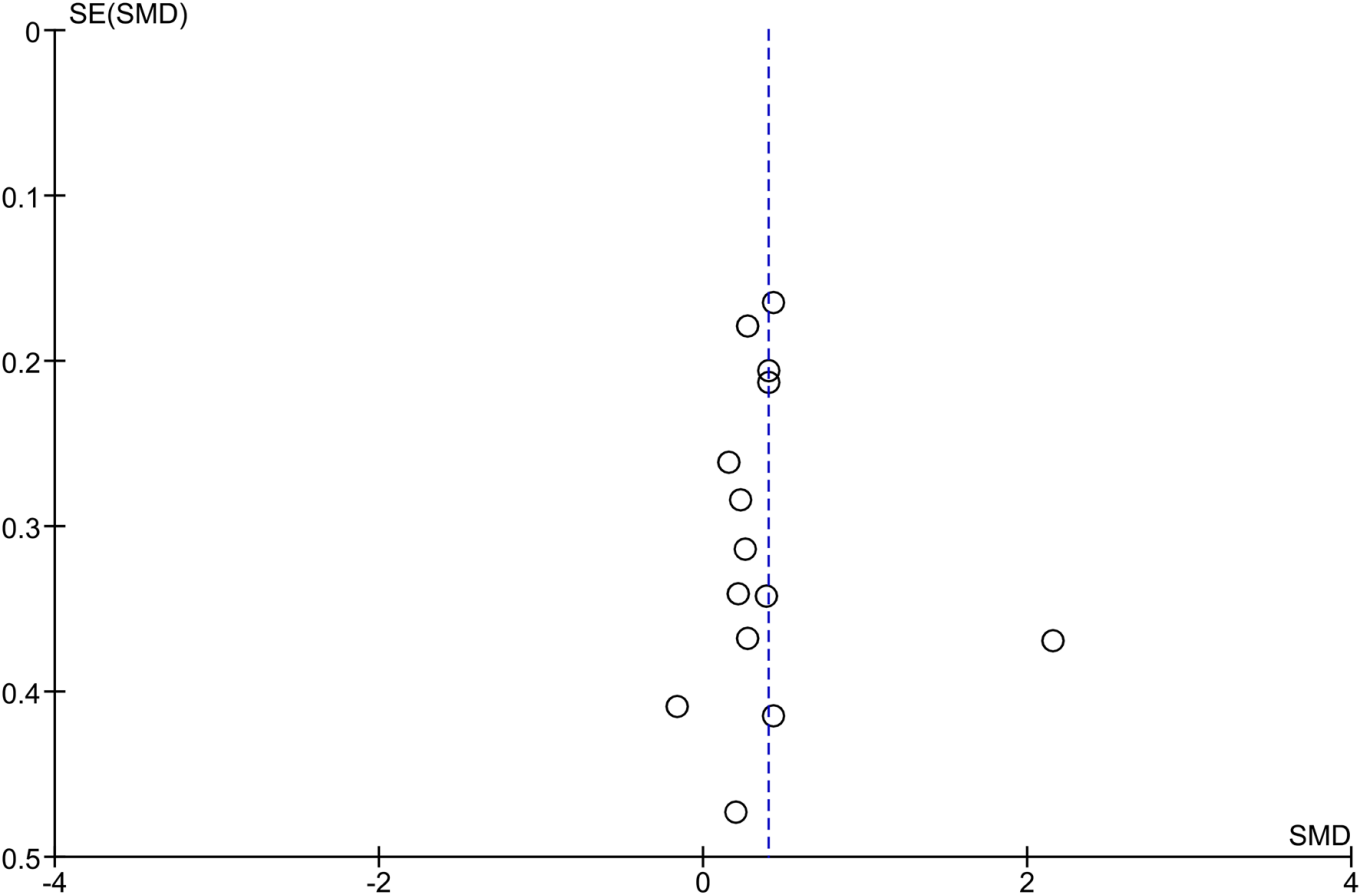
Funnel plot

Across the 14 studies heterogeneity was moderate (52%) and there was no evidence of publication bias (*p* = 0.7589) (Fig. 8).

### Study quality

**Fig. 9:**
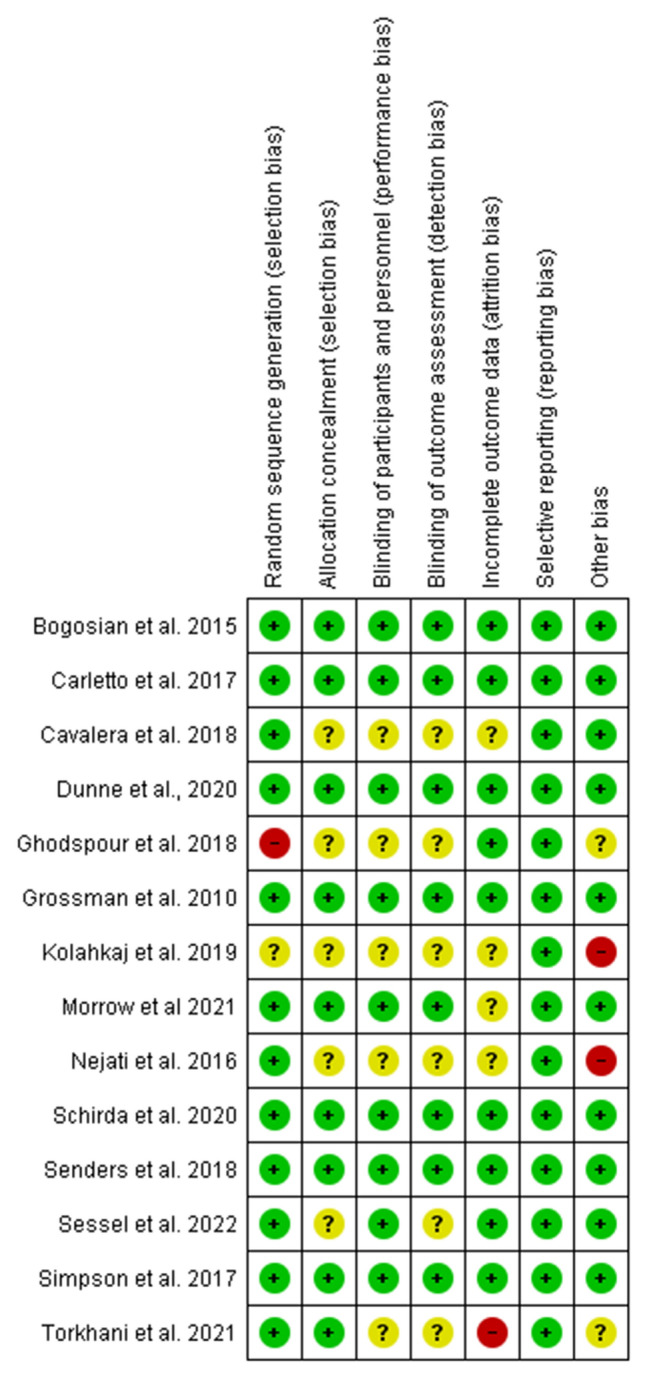
Risk of bias

There was no evidence of selective outcome reporting in any of the included studies. Most (*n* = 12 out of 14) described sequence generation, the majority (*n* = 9 out of 14) described allocation concealment, blinding procedures (*n* = 9 out of 14), and most (*n* = 9 out of 14) accounted for incomplete outcome reporting. Overall, half of included studies (*n* = 7 out of 14) were adjudged low risk of bias (Fig. 9).

### Adverse events

In one study, a participant undertaking MBSR reported an increase in neuropathic pain following the ‘raisin exercise’—an introductory MBI exercise, which involves exploring sensory experiences associated with seeing, touching, and tasting a raisin using mindful awareness [51]. In another study, a participant felt more anxious after a MBSR day retreat and a participant experienced muscle spasticity during a muscular relaxation activity [50]. Lastly, in one study, four participants experienced an MS relapse or hospitalization, however these events were deemed unrelated to the MBI [59].

## Discussion

### Main findings

Overall, 14 RCTs were eligible for inclusion in this systematic review and meta-analysis. Pooled results across all studies suggest MBIs effectively improve QoL among PwMS with moderate treatment effects (SMD = 0.40). However, when considering only those six studies employing an active comparator, pooled effects on QoL were smaller (SMD = 0.28). Most studies collected data at baseline, post-MBI, and a variable follow-up point ranging from 2 to 6 months. Across studies, a total of 937 PwMS participated. All MS phenotypes were included, the majority being relapsing remitting. Most studies tested group-based MBSR, or a tailored derivative, but there was a mix of face-to-face and online delivery. Most studies assessed QoL using MS-specific measures; effects sizes were larger in studies using a generic QoL measure (SMD = 0.61 vs 0.39). The largest effects were seen on mental QoL subscales (SMD = 0.70). Face-to-face MBIs had a non-significant trend toward larger treatment effects (SMD = 0.44) than online (SMD = 0.29). Study attrition and treatment adherence varied widely.

### Comparison with extant literature

No previous study has systematically assessed the RCT-based evidence specifically for efficacy of MBIs in PwMS for improving QoL. A previous systematic review and meta-analysis [65] of controlled trials (*n* = 21) testing MBI effects on depression, anxiety, stress, fatigue, and QoL among PwMS found a comparable effect on QoL when pooling just six studies (Hedge’s g = 0.22; 95% CI 0.0—0.45, *p* < 0.05), but did not examine differential effects relating to type of QoL measure or aspect of QoL under assessment. Another meta-analysis [66] of RCTs of psychosocial interventions for PwMS (total *n* = 1,617; mean age 47.18; 76% female; 71% relapsing remitting) assessing CBT [*n* = 6]; progressive muscular relaxation [*n* = 2]; self-management [*n* = 2]; mindfulness [*n* = 1]; motivational interviewing [*n* = 1]; coping skills [*n* = 1], reported significant small, but stable beneficial effects on overall (Cohen’s *d* = 0.308; 95% CI 0.143–0.473) and mental health-related QoL (*d* = 0.220; 95% CI 0.084–0.357). Treatment effects on physical health-related QoL were smaller and non-significant (*d* = 0.099; 95% CI 0.165–0.363). Intervention dose moderated outcomes, where higher therapy hours (range 3.5–50 h) increased effect sizes. This fits with data from non-MS populations, where MBI ‘dose’ (amount of home practice) mediates beneficial treatment effects, although minimum effective dose remains obscure and likely will vary [39]. In this current study, MBI dose (session attendance + home practice) was infrequently reported, but ranged from 16 to 66 h, with session attendance ranging from 60 to 95%, and home practice 29.2–38 min/day.

### Strengths and weaknesses of this study

We used recommended tools for carrying out our systematic review and meta-analysis, leaving our findings open to external scrutiny and audit. Our research team was multi-disciplinary (nursing, rehabilitation, family medicine, psychiatry, psychology, statistics). We included solely RCTs to collate the highest quality evidence for the use of MBIs to improve QoL in PwMS.

Our study was necessarily limited to include only those articles published in English. As the concepts underpinning mindfulness originally derive from Asia, it is possible we missed relevant literature (i.e., non-English language publications) on the use of this technology in diverse contexts, where participant characteristics, intervention acceptability and effects may differ somewhat. However, we found no statistical evidence of publication bias.

### Strengths and weaknesses of studies in this review

This study had several strengths. All studies in this systematic review and meta-analysis were RCTs. Six compared against an active comparator condition, attempting to minimize non-specific treatment effects, likely in a group-based complex intervention [67] such as MBIs [68]. An RCT is widely regarded as the best study design to minimize bias in the ‘hierarchy of evidence’ [69]. Although a wide range of participants took part in the studies in this review, mean participant age was relatively low (44.04), socioeconomic and educational statuses infrequently documented. Thus, very little is known about effects of MBIs among older PwMS, those with late onset disease, or with diverse social and educational backgrounds. Similarly, limited reporting on other factors known to impair (physical and mental health comorbidities, physical disability, cognitive impairment), stabilize or improve QoL in PwMS (e.g., ‘second generation’ DMD use [70]) limits somewhat the scope of analyses, whereas lack of biological outcome measurement (e.g., structural or functional MRI) limits somewhat interpretation of meaning in findings. In addition, regarding quality, although half of studies included in this review were deemed to have low risk of bias, reporting of study procedures, population characteristics, intervention components, and outcomes (particularly adherence) were not always consistent and room for improvement remains.

### Implications for research

MBIs effectively improve depression in PwMS [46], a factor strongly associated with reduced QoL in this population [18]. However, the impact of MBIs on other factors known to impair QoL in PwMS, such as cognitive impairment [17] should be assessed, as in general populations MBIs can improve aspects of cognitive function (working and autobiographical memory, cognitive flexibility, and meta-awareness) [71].

The factors that mediate or moderate effectiveness of MBIs in PwMS are not known. Feasibility work suggests important roles for acceptance, self-efficacy, and self-compassion [72]. Future research may examine the neurobiological mechanisms that underpin MBIs, as well as test a wider range of candidate factors in larger, powered samples of PwMS.

### Implications for clinical practice

MBIs appear to be a safe approach to improving QoL in PwMS, with the greatest benefits seen on mental QoL. Both face-to-face and online MBIs hold potential for effectiveness, though the small number of studies in this area makes drawing firm conclusions difficult. In pragmatic terms, online or virtual MBIs may now be preferrable to PwMS, given the ongoing context created by the COVID-19 pandemic, and may also help to address some of the inequalities PwMS face in accessing mental healthcare [73].

## Conclusions

MBIs effectively improve QoL in PwMS. The greatest benefits are on mental health-related QoL. However, more research is needed to characterize optimal formatting, mechanisms of action, and effects in PwMS with more diverse social, educational, and clinical backgrounds.

## Supplementary Information

Below is the link to the electronic supplementary material.

Supplementary file1 (DOCX 20 KB)

Supplementary file2 (DOC 90 KB)

Supplementary file3 (DOCX 27 KB)
