# Supplementary material for: A systematic review and meta-analysis exploring the efficacy of mindfulness-based interventions on quality of life in people with multiple sclerosis
Source: J Neurol. 2022 Nov 9;270(2):726–45. doi: 10.1007/s00415-022-11451-x (PMC9643979; doi:10.1007/s00415-022-11451-x)
Supplement: Supplementary file 1 — Supplementary file1 (DOCX 20 KB) [file 415_2022_11451_MOESM1_ESM.docx]

Database: Ovid MEDLINE(R) and Epub Ahead of Print, In-Process, In-Data-Review & Other Non-Indexed Citations, Daily and Versions(R) <1946 to April 05, 2021>

Search Strategy:

--------------------------------------------------------------------------------

1 exp Multiple Sclerosis/ (60789)

2 exp Neuromyelitis Optica/ (3151)

3 exp Multiple Sclerosis, Chronic Progressive/ (2091)

4 Multiple Sclerosis, Relapsing-Remitting/ (6540)

5 "disseminated sclerosis".mp. (634)

6 devic.mp. (205)

7 "acute disseminated encephalomyelitis".mp. (1844)

8 encephalomyelitis.mp. (26266)

9 "multiple sclerosis".mp. (87157)

10 "neuromyelitis optica".mp. (4868)

11 "optic neuritis".mp. (8684)

12 "transverse myelitis".mp. (2330)

13 demyelinat*.mp. (38139)

14 myelitis.mp. (6933)

15 ((clinically or radiologically) adj1 isolated syndrome*).mp. (1781)

16 (demyelinating adj1 (disease* or disorder*)).mp. (18958)

17 or/1-16 (137792)

18 exp Mindfulness/ (3890)

19 exp Meditation/ (3011)

20 exp Breathing Exercises/ (3761)

21 (MBSR or MBCT).mp. (1158)

22 relaxation.mp. (130870)

23 (breathing adj1 (exercis* or techniqu*)).mp. (4813)

24 vipassana.mp. (61)

25 yoga.mp. (5678)

26 mindful*.mp. (11817)

27 meditat*.mp. (7625)

28 or/18-27 (153830)

29 17 and 28 (832)

30 limit 29 to (english language and yr="1980 -Current") (800)

***************************

Database: Embase Classic+Embase <1947 to 2021 Week 13>

Search Strategy:

--------------------------------------------------------------------------------

1 exp multiple sclerosis/ (142366)

2 exp myelooptic neuropathy/ (9845)

3 "disseminated sclerosis".mp. (961)

4 devic.mp. (446)

5 "acute disseminated encephalomyelitis".mp. (4090)

6 encephalomyelitis.mp. (39497)

7 "multiple sclerosis".mp. (155619)

8 "neuromyelitis optica".mp. (8768)

9 "optic neuritis".mp. (15184)

10 "transverse myelitis".mp. (4492)

11 demyelinat*.mp. (67865)

12 myelitis.mp. (13217)

13 ((clinically or radiologically) adj1 isolated syndrome*).mp. (4047)

14 (demyelinating adj1 (disease* or disorder*)).mp. (24069)

15 or/1-14 (234024)

16 exp mindfulness/ (10202)

17 exp meditation/ (8726)

18 exp breathing exercise/ (9049)

19 (MBSR or MBCT).mp. (1780)

20 relaxation.mp. (162383)

21 (breathing adj1 (exercis* or techniqu*)).mp. (10537)

22 vipassana.mp. (87)

23 exp yoga/ (8626)

24 yoga.mp. (10429)

25 mindful*.mp. (17499)

26 meditat*.mp. (12473)

27 or/16-26 (200752)

28 15 and 27 (1495)

29 limit 28 to (english language and yr="1980 -Current") (1392)

***************************

Database: EBM Reviews - Cochrane Central Register of Controlled Trials <February 2021>

Search Strategy:

--------------------------------------------------------------------------------

1 exp Multiple Sclerosis/ (3590)

2 exp Neuromyelitis Optica/ or myelooptic neuropath*.mp. (208)

3 exp Multiple Sclerosis, Chronic Progressive/ (263)

4 Multiple Sclerosis, Relapsing-Remitting/ (899)

5 "disseminated sclerosis".mp. (2)

6 devic.mp. (16)

7 "acute disseminated encephalomyelitis".mp. (31)

8 encephalomyelitis.mp. (253)

9 "multiple sclerosis".mp. (11053)

10 "neuromyelitis optica".mp. (241)

11 "optic neuritis".mp. (495)

12 "transverse myelitis".mp. (60)

13 demyelinat*.mp. (1339)

14 myelitis.mp. (215)

15 ((clinically or radiologically) adj1 isolated syndrome*).mp. (230)

16 (demyelinating adj1 (disease* or disorder*)).mp. (506)

17 or/1-16 (12357)

18 exp Mindfulness/ (918)

19 exp Meditation/ (614)

20 exp Breathing Exercises/ (856)

21 (MBSR or MBCT).mp. (1307)

22 relaxation.mp. (12348)

23 (breathing adj1 (exercis* or techniqu*)).mp. (2845)

24 vipassana.mp. (16)

25 yoga.mp. (3600)

26 mindful*.mp. (6110)

27 meditat*.mp. (3196)

28 or/18-27 (23738)

29 17 and 28 (309)

30 limit 29 to (yr="1980 -Current" and english language) (184)

***************************

Database: APA PsycInfo <1806 to March Week 5 2021>

Search Strategy:

--------------------------------------------------------------------------------

1 exp Multiple Sclerosis/ (13172)

2 (neuromyelitis optica or myelooptic neuropath*).mp. (1015)

3 "disseminated sclerosis".mp. (33)

4 devic.mp. (18)

5 "acute disseminated encephalomyelitis".mp. (314)

6 encephalomyelitis.mp. (2324)

7 "multiple sclerosis".mp. (16776)

8 "optic neuritis".mp. (813)

9 "transverse myelitis".mp. (328)

10 demyelinat*.mp. (5686)

11 myelitis.mp. (919)

12 ((clinically or radiologically) adj1 isolated syndrome*).mp. (501)

13 (demyelinating adj1 (disease* or disorder*)).mp. (1389)

14 or/1-13 (21508)

15 exp Mindfulness/ (10506)

16 exp Mindfulness-Based Interventions/ (1393)

17 exp Meditation/ (4910)

18 (MBSR or MBCT).mp. (1438)

19 exp Relaxation/ (2529)

20 exp Relaxation Therapy/ (3562)

21 relaxation.mp. (17344)

22 (breathing adj1 (exercis* or techniqu*)).mp. (585)

23 vipassana.mp. (178)

24 exp Yoga/ (1954)

25 yoga.mp. (3268)

26 mindful*.mp. (18739)

27 meditat*.mp. (10531)

28 or/15-27 (43191)

29 14 and 28 (233)

30 limit 29 to (english language and yr="1980 -Current") (217)

***************************

Database: AMED (Allied and Complementary Medicine) <1985 to January 2021>

Search Strategy:

--------------------------------------------------------------------------------

1 exp Multiple sclerosis/ (1880)

2 "disseminated sclerosis".mp. (0)

3 devic.mp. (1)

4 "acute disseminated encephalomyelitis".mp. (4)

5 encephalomyelitis.mp. (152)

6 "multiple sclerosis".mp. (2221)

7 "neuromyelitis optica".mp. (4)

8 "optic neuritis".mp. (13)

9 "transverse myelitis".mp. (23)

10 demyelinat*.mp. (124)

11 myelitis.mp. (41)

12 ((clinically or radiologically) adj1 isolated syndrome*).mp. (6)

13 (demyelinating adj1 (disease* or disorder*)).mp. (41)

14 or/1-13 (2477)

15 Mindful*.mp. (591)

16 exp Meditation/ (455)

17 exp Breathing exercises/ (307)

18 (MBSR or MBCT).mp. (54)

19 exp Relaxation/ (1108)

20 relaxation.mp. (2615)

21 (breathing adj1 (exercis* or techniqu*)).mp. (479)

22 vipassana.mp. (7)

23 exp Yoga/ (834)

24 yoga.mp. (974)

25 meditat*.mp. (800)

26 or/15-25 (4870)

27 14 and 26 (45)

28 limit 27 to (english summary and yr="1980 -Current") (1)

***************************

Cinahl Strategy:

| **Search ID#** | **Search Terms** | **Search Options** | **Results** |
| --- | --- | --- | --- |
| S30 | S17 AND S29 | Limiters - Published Date: 19800101-20211231; English Language  Expanders - Apply equivalent subjects  Search modes - Boolean/Phrase | 451 |
| S29 | S18 OR S19 OR S20 OR S21 OR S22 OR S23 OR S24 OR S25 OR S26 OR S27 OR S28 | Expanders - Apply equivalent subjects  Search modes - Boolean/Phrase | 46702 |
| S28 | TX meditat* | Expanders - Apply equivalent subjects  Search modes - Boolean/Phrase | 7805 |
| S27 | TX mindful* | Expanders - Apply equivalent subjects  Search modes - Boolean/Phrase | 11286 |
| S26 | TX yoga | Expanders - Apply equivalent subjects  Search modes - Boolean/Phrase | 12810 |
| S25 | (MH "Yoga+") | Expanders - Apply equivalent subjects  Search modes - Boolean/Phrase | 9700 |
| S24 | TX vipassana | Expanders - Apply equivalent subjects  Search modes - Boolean/Phrase | 30 |
| S23 | TX (breathing N1 (exercis* or techniqu*)) | Expanders - Apply equivalent subjects  Search modes - Boolean/Phrase | 3257 |
| S22 | TX relaxation | Expanders - Apply equivalent subjects  Search modes - Boolean/Phrase | 19493 |
| S21 | TX (MBSR or MBCT) | Expanders - Apply equivalent subjects  Search modes - Boolean/Phrase | 681 |
| S20 | (MH "Breathing Exercises+") | Expanders - Apply equivalent subjects  Search modes - Boolean/Phrase | 2550 |
| S19 | (MH "Meditation") | Expanders - Apply equivalent subjects  Search modes - Boolean/Phrase | 5437 |
| S18 | (MH "Mindfulness") | Expanders - Apply equivalent subjects  Search modes - Boolean/Phrase | 5706 |
| S17 | S1 OR S2 OR S3 OR S4 OR S5 OR S6 OR S7 OR S8 OR S9 OR S10 OR S11 OR S12 OR S13 OR S14 OR S15 OR S16 | Expanders - Apply equivalent subjects  Search modes - Boolean/Phrase | 34871 |
| S16 | TX (demyelinating N1 (disease* or disorder*)) | Expanders - Apply equivalent subjects  Search modes - Boolean/Phrase | 3026 |
| S15 | TX ((clinically or radiologically) N1 isolated syndrome*) | Expanders - Apply equivalent subjects  Search modes - Boolean/Phrase | 434 |
| S14 | (MH "Demyelinating Diseases+") | Expanders - Apply equivalent subjects  Search modes - Boolean/Phrase | 26092 |
| S13 | TX myelitis | Expanders - Apply equivalent subjects  Search modes - Boolean/Phrase | 1495 |
| S12 | TX demyelinat* | Expanders - Apply equivalent subjects  Search modes - Boolean/Phrase | 5605 |
| S11 | TX "transverse myelitis" | Expanders - Apply equivalent subjects  Search modes - Boolean/Phrase | 587 |
| S10 | TX "optic neuritis" | Expanders - Apply equivalent subjects  Search modes - Boolean/Phrase | 995 |
| S9 | TX "neuromyelitis optica" | Expanders - Apply equivalent subjects  Search modes - Boolean/Phrase | 1004 |
| S8 | TX "multiple sclerosis" | Expanders - Apply equivalent subjects  Search modes - Boolean/Phrase | 25842 |
| S7 | TX encephalomyelitis | Expanders - Apply equivalent subjects  Search modes - Boolean/Phrase | 1889 |
| S6 | (MH "Encephalomyelitis, Acute Disseminated") | Expanders - Apply equivalent subjects  Search modes - Boolean/Phrase | 293 |
| S5 | TX "acute disseminated encephalomyelitis" | Expanders - Apply equivalent subjects  Search modes - Boolean/Phrase | 461 |
| S4 | TX devic | Expanders - Apply equivalent subjects  Search modes - Boolean/Phrase | 109 |
| S3 | TX "disseminated sclerosis" | Expanders - Apply equivalent subjects  Search modes - Boolean/Phrase | 4 |
| S2 | (MH "Neuromyelitis Optica") | Expanders - Apply equivalent subjects  Search modes - Boolean/Phrase | 464 |
| S1 | (MH "Multiple Sclerosis+") | Expanders - Apply equivalent subjects  Search modes - Boolean/Phrase | 20637 |
