# Supplementary material for: A systematic review and meta-analysis exploring the efficacy of mindfulness-based interventions on quality of life in people with multiple sclerosis
Source: J Neurol. 2022 Nov 9;270(2):726–45. doi: 10.1007/s00415-022-11451-x (PMC9643979; doi:10.1007/s00415-022-11451-x)
Supplement: Supplementary file 2 — Supplementary file2 (DOC 90 KB) [file 415_2022_11451_MOESM2_ESM.doc]

Data Extraction Sheet v.1 Simpson et al.

| **Bibliographic details** | |
| --- | --- |
| **Authors** |  |
| **Year** |  |
| **Country** |  |
| **Citation** |  |
| **Title (identifying study as an RCT y/n?)** |  |
| **Structured abstract** |  |
| **References identified from reference list** | Yes/no  If yes, please provide details: |

| **Study** | |
| --- | --- |
| **Study aims and objectives** |  |
| **Study design** |  |
| **Comparator group** |  |
| **Statistical methods** |  |
| **Power calculation** |  |
| **Inclusion criteria** |  |
| **Exclusion criteria** |  |
| **Stopping criteria** |  |
| **Setting/location where data collected** |  |
| **Trial protocol/ registration** |  |
| **Ethical approval** |  |
| **Funding details y/n?** |  |

| **Population** | | |
| --- | --- | --- |
|  | **Intervention group** | **Control /comparison group** |
| **Sample size** |  |  |
| **Recruited from where?** |  |  |
| **Age** |  |  |
| **Sex** |  |  |
| **Socioeconomic status** |  |  |
| **Ethnicity** |  |  |
| **Marital status** |  |  |
| **Living Arrangements** |  |  |
| **Educational status** |  |  |
| **Employment status** |  |  |
| **Disease phenotype** |  |  |
| **Use of disease modifying drugs** |  |  |
| **Time since diagnosis** |  |  |
| **Disability level** |  |  |
| **Cognitive impairment** |  |  |
| **Comorbid anxiety (% on drug treatment)** |  |  |
| **Comorbid depression (% on drug treatment)** |  |  |
| **Other comorbidities** |  |  |

| **Intervention** | |
| --- | --- |
| **Definition** |  |
| **Course content** |  |
| **Tailored to population? (If yes, describe)** |  |
| **Fidelity to treatment assessed y/n (if so, how)** |  |
| **Course completion criteria** |  |
| **Mode of delivery (face-to-face, internet etc)** |  |
| **Duration & frequency** |  |
| **Instructor characteristics** |  |
| **No. of participants per group** |  |
| **Intervention materials** |  |
| **Intervention location** |  |
| **Cost to participants** |  |
| **Cost effectiveness** |  |
| **Transport issues** | . |
| **Family/carer involvement** |  |
| **Other (specify)** |  |
| **Intervention for control group** | |
| **Provide details:** | |

| **Outcomes** | | |
| --- | --- | --- |
|  | **Intervention group** | **Control /comparison group** |
| **Feasibility outcomes**   - **Recruitment (to pre-defined target y/n?)** - **Randomisation** - **Retention** - **Adherence (classes attendance/ home-practice completion)** - **Follow-up (when?)** - **Reasons accounting for attrition reported** - **Missing data** |  |  |
| **CONSORT flow diagram y/n** |  |  |
| **Adverse events reported y/n? (specify)** |  |  |
| **Standardised outcomes measures (specify)** |  |  |
| **Study-specific outcomes measures (provide details)** |  |  |
| **Sub-group analyses** |  |  |
| **Other outcomes measured (provide details)** |  |  |
| **No. of data collection time points** |  |  |

| **Limitations/conclusions/comments** | |
| --- | --- |
| **Limitations noted by the authors** |  |
| **Authors’ conclusions** |  |
| **Reviewer’s comments** |  |

| **Risk of bias assessment (High/unclear/low)** | |
| --- | --- |
| Random sequence generation (selection bias) |  |
| Allocation concealment (selection bias) |  |
| Blinding of assessors (performance bias) |  |
| Blinding of outcome assessment (detection bias) (patient reported outcomes) |  |
| Incomplete outcome data addressed (attrition bias) |  |
| Selective outcome reporting (reporting bias) |  |
| Other sources of bias (i.e. baseline bias) |  |
