# Supplementary material for: A systematic review and meta-analysis exploring the efficacy of mindfulness-based interventions on quality of life in people with multiple sclerosis
Source: J Neurol. 2022 Nov 9;270(2):726–45. doi: 10.1007/s00415-022-11451-x (PMC9643979; doi:10.1007/s00415-022-11451-x)
Supplement: Supplementary file 3 — Supplementary file3 (DOCX 27 KB) [file 415_2022_11451_MOESM3_ESM.docx]

RoB - Grossman et al. 2010

| **Risk of bias assessment (High/unclear/low)** | |
| --- | --- |
| Random sequence generation (selection bias) | Low – Baseline assessments prior to randomisation. PI randomised blind, using [www.randomizer.org](http://www.randomizer.org) in blocks of 4 - 6 |
| Allocation concealment (selection bias) | Low – PI sent allocation list to co-ordinator who informed participants in writing of their assignment. This was then re-checked by PI, no deviations found |
| Blinding of assessors (performance bias) | Low – Investigators blinded to assignment |
| Blinding of outcome assessment (detection bias) (patient reported outcomes) | Low – All PRO measures were entered into a database by blinded personnel |
| Incomplete outcome data addressed (attrition bias) | Low – Consort flow diagram included in report with n randomized, n analysed etc. ITT employed. Missing data imputed by multiple linear regression that adjusted for age, gender, and disease progression |
| Selective outcome reporting (reporting bias) | Low – All pre-specified outcomes were reported |
| Other sources of bias (i.e. baseline bias) | Low – Well conducted and reported study |

RoB – Bogosian et al. 2015

| **Risk of bias assessment (High/unclear/low)** | |
| --- | --- |
| Random sequence generation (selection bias) | Low – Randomisation took place once cohort of 10 participants consented, screened and baseline data collected. Independent unit at KCL Clinical Trials Unit (CTU) handled randomisation, with fixed block sizes of 2 |
| Allocation concealment (selection bias) | Low – As above. Then CTU sent assignment list to PI |
| Blinding of assessors (performance bias) | Low – Trial assessor blinded to allocation |
| Blinding of outcome assessment (detection bias) (patient reported outcomes) | Low – Statistician, health economist blinded to assignment |
| Incomplete outcome data addressed (attrition bias) | Low – Consort flow diagram included in report with n randomized, n analysed etc. ITT employed. Informative missingness processes explored by sensitivity analysis. Missing baseline variables handled using the missing indicator method |
| Selective outcome reporting (reporting bias) | Low – All pre-specified outcomes were reported |
| Other sources of bias (i.e. baseline bias) | Low – Well conducted and reported study |

RoB – Nejati et al. 2016

| **Risk of bias assessment (High/unclear/low)** | |
| --- | --- |
| Random sequence generation (selection bias) | Low – Each participant’s names placed on slip of paper, mixed and drawn randomly |
| Allocation concealment (selection bias) | Unclear - Paper only states that participants were selected using a random sampling method |
| Blinding of assessors (performance bias) | Unclear – Not reported in the paper |
| Blinding of outcome assessment (detection bias) (patient reported outcomes) | Unclear – Not reported in the paper |
| Incomplete outcome data addressed (attrition bias) | Unclear – Attrition not reported, nor numbers included in analyses or details regarding missing data |
| Selective outcome reporting (reporting bias) | Low – All pre-specified outcomes were reported |
| Other sources of bias (i.e. baseline bias) | High – Paper states study population based on convenience sampling |

RoB – Simpson et al. 2017

| **Risk of bias assessment (High/unclear/low)** | |
| --- | --- |
| Random sequence generation (selection bias) | Low – Post-baseline measures an independent statistician undertook block randomisation and sequence generation |
| Allocation concealment (selection bias) | Low – Blinded research staff undertook treatment allocation |
| Blinding of assessors (performance bias) | Low – Research staff were blinded to treatment allocation and participant ID |
| Blinding of outcome assessment (detection bias) (patient reported outcomes) | Low – Anonymous data was collected by a blinded research assistant |
| Incomplete outcome data addressed (attrition bias) | Low – Detailed reporting of missing data, no imputation. Consort flow diagram and details accounting for participant drop-out |
| Selective outcome reporting (reporting bias) | Low – All pre-specified outcomes were reported |
| Other sources of bias (i.e. baseline bias) | Low – Well conducted and reported study |

RoB – Carletto et al. 2017

| **Risk of bias assessment (High/unclear/low)** | |
| --- | --- |
| Random sequence generation (selection bias) | Low – Randomly assigned on 1:1 ratio using a blockwise randomisation sequence |
| Allocation concealment (selection bias) | Low – Sequence determined by an independent researcher blinded to initial assessment. Study co-ordinator communicated assignment to participants |
| Blinding of assessors (performance bias) | Low – Clinical Psychologists performing assessments were blinded to participant ID |
| Blinding of outcome assessment (detection bias) (patient reported outcomes) | Low – Clinical Psychologists performing assessments were blinded to participant ID |
| Incomplete outcome data addressed (attrition bias) | Low – Both PP and ITT performed - ITT explored missing data – data imputation was used for two participants. Consort flow diagram detailing numbers analysed and dropping out. Comparison between completers and dropouts baseline measures and socio-demographics undertaken. |
| Selective outcome reporting (reporting bias) | Low – All pre-specified outcomes were reported |
| Other sources of bias (i.e. baseline bias) | Low – Well conducted and reported study |

RoB – Cavalera et al. 2018

| **Risk of bias assessment (High/unclear/low)** | |
| --- | --- |
| Random sequence generation (selection bias) | Low – Participants were randomly assigned 1:1 to MBI and control using [www.random.org](http://www.random.org) |
| Allocation concealment (selection bias) | Unclear – Paper only states that participants were randomly assigned to MBI and control |
| Blinding of assessors (performance bias) | Unclear – Not reported in the paper |
| Blinding of outcome assessment (detection bias) (patient reported outcomes) | Unclear – Not reported in the paper |
| Incomplete outcome data addressed (attrition bias) | Unclear – Although consort flow diagram included, detailing attrition, reasons accounting for this were insufficiently described. No mention of missing data |
| Selective outcome reporting (reporting bias) | Low – All pre-specified outcomes were reported |
| Other sources of bias (i.e. baseline bias) | Low – Generally well conducted and reported study |

RoB – Senders et al. 2018

| **Risk of bias assessment (High/unclear/low)** | |
| --- | --- |
| Random sequence generation (selection bias) | Low – Statistician generated randomization scheme stratified by baseline PSS scores with a block size of four (SPSS random number generator) |
| Allocation concealment (selection bias) | Low – Randomisation scheme maintained by individual external and blinded to study. Allocation concealed from all study staff |
| Blinding of assessors (performance bias) | Low – Baseline data collected prior to randomisation – PI, statistician and personnel performing data entry were blinded to group assignment |
| Blinding of outcome assessment (detection bias) (patient reported outcomes) | Low – Baseline data collected prior to randomisation – PI, statistician and personnel performing data entry were blinded to group assignment |
| Incomplete outcome data addressed (attrition bias) | Low – Low – Consort flow diagram detailing reasons accounting for attrition and numbers analysed. |
| Selective outcome reporting (reporting bias) | Low – All pre-specified outcomes were reported |
| Other sources of bias (i.e. baseline bias) | Low – Well conducted and reported study |

RoB – Ghodspour et al. 2018

| **Risk of bias assessment (High/unclear/low)** | |
| --- | --- |
| Random sequence generation (selection bias) | High – ‘Purposive’ sampling before unclear method of random allocation to groups |
| Allocation concealment (selection bias) | Unclear– Not described |
| Blinding of assessors (performance bias) | Unclear– Not described |
| Blinding of outcome assessment (detection bias) (patient reported outcomes) | Unclear– Not described |
| Incomplete outcome data addressed (attrition bias) | Low – Attrition and reasons accounting for discussed. |
| Selective outcome reporting (reporting bias) | Low – All pre-specified outcomes were reported |
| Other sources of bias (i.e. baseline bias) | Unclear– Generally, poor reporting and no reference to CONSORT |

RoB – Torkhani et al., 2021

| **Risk of bias assessment (High/unclear/low)** | |
| --- | --- |
| Random sequence generation (selection bias) | Low- Randomization list generated |
| Allocation concealment (selection bias) | Low- Treatment condition unknown to coordinator to ensure concealment of allocation |
| Blinding of assessors (performance bias) | Unclear- Not enough detail surrounding whether those evaluating outcomes were blinded |
| Blinding of outcome assessment (detection bias) (patient reported outcomes) | Unclear- No details surrounding patients being blinded |
| Incomplete outcome data addressed (attrition bias) | High- No mention of attrition or reasons |
| Selective outcome reporting (reporting bias) | Low- All pre-specified outcomes reported |
| Other sources of bias (i.e. baseline bias) | Unclear- Recruitment procedure open to selection bias |

RoB – Sesel et al., 2022

| **Risk of bias assessment (High/unclear/low)** | |
| --- | --- |
| Random sequence generation (selection bias) | Low- Participants were then randomized using computer-generated random numbers to receive either the MBI or waitlist. |
| Allocation concealment (selection bias) | Unclear-Not enough details on the allocation—only Participants were stratified according to history of recurrent depression (Y/N) to ensure a roughly equal representation in each group |
| Blinding of assessors (performance bias) | Low- A list of participant identification numbers identifying those with a history of Major Depression (two or more episodes) were sent to a researcher not involved in the assessment or treatment of participants |
| Blinding of outcome assessment (detection bias) (patient reported outcomes) | Unclear- No details surrounding patients being blinded |
| Incomplete outcome data addressed (attrition bias) | Low- Attrition reasons given |
| Selective outcome reporting (reporting bias) | Low- All prespecified outcomes reported |
| Other sources of bias (i.e. baseline bias) | Low—Well conducted and reported study |

RoB – Schirda et al., 2020

| **Risk of bias assessment (High/unclear/low)** | |
| --- | --- |
| Random sequence generation (selection bias) | Low- The randomization sequence was generated by the principal investigator using a random number generator program (randomization.com) with a random block size of three or six |
| Allocation concealment (selection bias) | Low -Assignment concealed in ‘individual envelopes’ |
| Blinding of assessors (performance bias) | Low- All assessment sessions were completed by blinded study personnel. |
| Blinding of outcome assessment (detection bias) (patient reported outcomes) | Low- All assessment sessions were carried out by blinded study personnel’ |
| Incomplete outcome data addressed (attrition bias) | Low- Drop out reasons provided |
| Selective outcome reporting (reporting bias) | Low- All pre-specified outcomes reported |
| Other sources of bias (i.e. baseline bias) | Low- Well conducted and reported study |

RoB – Dunne et al., 2021

| **Risk of bias assessment (High/unclear/low)** | |
| --- | --- |
| **Random sequence generation** (selection bias) | Low- Using blocks of three dummy codes for the groups, participants were randomly allocated to M4MS, Chair Yoga, or the wait-list control group |
| **Allocation concealment** (selection bias) | Low- Assignment of each group was blinded to the research personnel’ |
| **Blinding of assessors** (performance bias) | Low- Assignment to each group was blinded to the research personnel primarily involved in screening participants, recording and analyzing preliminary data. |
| **Blinding of outcome assessment** (detection bias) (patient reported outcomes) | Low- All assessment sessions were carried out by blinded study personnel primarily involved in screening participants, recording and analysing preliminary data’ |
| **Incomplete outcome data addressed** (attrition bias) | Low- Attrition reasons provided |
| **Selective outcome reporting** (reporting bias) | Low- All pre-specified outcomes reported |
| **Other sources of bias** (i.e. baseline bias) | Low- Well conducted and reported study |

RoB – Morrow et al., 2021

| **Risk of bias assessment (High/unclear/low)** | |
| --- | --- |
| **Random sequence generation** (selection bias) | Low- Using a computer-based random number generator, subjects were assigned to the intervention and the usual care (control) groups. |
| **Allocation concealment** (selection bias) | Low- The treating physician and research associates who assessed subjects were all blinded to the group assignment |
| **Blinding of assessors** (performance bias) | Low- The treating physician and research associates who assessed subjects were all blinded to the group assignment |
| **Blinding of outcome assessment** (detection bias) (patient reported outcomes) | Low- Not really possible to blind in this case |
| **Incomplete outcome data addressed** (attrition bias) | Unclear- Due to non-completers being excluded |
| **Selective outcome reporting** (reporting bias) | Low- All pre-specified outcomes reported |
| **Other sources of bias** (i.e. baseline bias) | Low- Well conducted and reported study |

RoB – Kolahkaj et al., 2019

| **Risk of bias assessment (High/unclear/low)** | |
| --- | --- |
| **Random sequence generation** (selection bias) | Unclear- Not enough details on who did randomization and how |
| **Allocation concealment** (selection bias) | Unclear- Not reported in paper |
| **Blinding of assessors** (performance bias) | Unclear- Not reported in paper |
| **Blinding of outcome assessment** (detection bias) (patient reported outcomes) | Unclear- Not reported in paper |
| **Incomplete outcome data addressed** (attrition bias) | Unclear- Not reported in paper |
| **Selective outcome reporting** (reporting bias) | Low- All pre-specified outcomes reported |
| **Other sources of bias** (i.e. baseline bias) | High- Convenience sampling |
